# Effects of awake prone position vs. usual care on acute hypoxemic respiratory failure in patients with COVID-19: A systematic review and meta-analysis of randomized controlled trials

**DOI:** 10.3389/fmed.2023.1120837

**Published:** 2023-04-04

**Authors:** Qing Peng, Sheng Yang, Yu Zhang, Wenjie Zhao, Man Hu, Bo Meng, Huanhuan Ni, Lingfeng Min, Jiangquan Yu, Yongxiang Wang, Liang Zhang

**Affiliations:** ^1^Department of Orthopedics, Clinical Medical College of Yangzhou University, Yangzhou, China; ^2^Department of Orthopedics, Graduate School of Dalian Medical University, Dalian, China; ^3^Department of Anesthesiology, Children's Hospital of Nanjing Medical University, Nanjing, China; ^4^Department of Respiratory, Clinical Medical College of Yangzhou University, Yangzhou, China; ^5^Department of Critical Care Medicine, Clinical Medical College of Yangzhou University, Yangzhou, China; ^6^Department of Orthopedics, Regenerative Medicine Engineering Technology Research Center of Yangzhou, Yangzhou, China

**Keywords:** COVID-19, hypoxemic respiratory failure, ARDS, oxygenation, intubation rate, mortality, awake prone positioning

## Abstract

**Background:**

Previous studies have shown that an awake prone position may be beneficial for the treatment of acute respiratory distress syndrome (ARDS) or acute hypoxic respiratory failure (AHRF) in patients with COVID-19, but the results are not consistent, especially in terms of oxygenation outcomes and intubation rate. This systematic review and meta-analysis assessed the effects of the awake prone position on AHRF in patients with COVID-19 with all randomized controlled trials (RCTs).

**Methods:**

An extensive search of online databases, including MEDLINE, Embase, Web of Science, and Cochrane Central Register of Controlled Trials from 1 December 2019 to 30 October 2022, with no language restrictions was performed. This systematic review and meta-analysis are based on the PRISMA statement. We only included RCTs and used the Cochrane risk assessment tool for quality assessment.

**Results:**

Fourteen RCTs fulfilled the selection criteria, and 3,290 patients were included. A meta-analysis found that patients in the awake prone position group had more significant improvement in the SpO_2_/FiO_2_ ratio [mean difference (MD): 29.76; 95% confidence interval (CI): 1.39–48.13; *P* = 0.001] compared with the usual care. The prone position also reduced the need for intubation [odd ratio (OR): 0.72; 95% CI: 0.61 to 0.84; *P* < 0.0001; *I*^2^ = 0%]. There was no significant difference in mortality, hospital length of stay, incidence of intensive care unit (ICU) admission, and adverse events between the two groups.

**Conclusion:**

The awake prone position was a promising intervention method, which is beneficial to improve the oxygenation of patients with ARDS or AHRF caused by COVID-19 and reduce the need for intubation. However, the awake prone position showed no obvious advantage in mortality, hospital length of stay, incidence of ICU admission, and adverse events.

**Systematic review registration:**

International Prospective Register of Systematic Reviews (PROSPERO), identifier: CRD42022367885.

## Introduction

During the early phase of the COVID-19 epidemic, the number of patients soared, which brought great challenges to the hospital resources and intensive care unit (ICU) ability. The awake prone position is widely recommended for its potential benefits such as ease of implementation, low risk, and reduced ICU admission requirement (1, 2). Prone position, non-invasive mechanical ventilation, and high-flow oxygen are regarded as feasible and safe interventions in acute hypoxic respiratory failure (AHRF) or acute respiratory distress syndrome (ARDS) (3, 4). The first proposal suggested that the prone position should be used to treat COVID-19, and they believe that the prone position can reduce the need for endotracheal intubation and invasive mechanical ventilation (5).

Previous studies have shown that the awake prone position can improve oxygenation and reduce mortality in patients with ARDS (6, 7). Prone position can increase alveolar ventilation, reduce shunt, and improve ventilation/perfusion ratio (8). The prone position can also recruit the alveoli in the gravity-dependent area (9) and reduce ventilator-associated lung injury (10). Although the prone position is more and more widely used, there is no unified conclusion about its effect on COVID-19 patients with AHRF. On the other hand, the prone position may lead to some negative effects such as reducing comfort and increasing diaphragm fraction (11).

Some observational studies also found that the awake prone position can improve oxygenation in patients with ARDS or AHRF caused by COVID-19 (12–15). However, the effect on intubation rate and mortality of patients has not reached a unified and clear conclusion. In addition, some randomized controlled trials (RCTs) have come to contradictory conclusions in these areas. In the recent three systematic reviews and meta-analyses (16–18), Li et al. reported that the awake prone position can reduce the need for intubation, but have no significant effect on mortality in COVID-19-associated patients with AHRF (16). Kang et al. found that the prone position can reduce the intubation rate and mortality of patients (17). Fazzini et al. reported that the prone position can improve oxygenation and mortality, but show no significant effect on intubation rate and ICU admission (18).

A systematic review and meta-analysis of RCTs showed that the prone position could effectively improve oxygenation and reduce the intubation rate in patients with COVID-19 (19). However, this study has some limitations. First, the number of studies that can be included is small and the heterogeneity is high. Second, this study did not compare whether there is a significant improvement in oxygenation before and after prone position intervention. In addition, after the completion of the system review, four new RCTs (20–23) were published recently. The purpose of this study was to further explore the clinical outcome of awake prone position on patients with ARDS or ARHF caused by COVID-19. Primary outcomes included oxygenation, intubation rate, and secondary outcomes included mortality, hospital length of stay (LOS), ICU admission, and incidence of adverse events.

## Methods

This systematic review and meta-analysis are based on the PRISMA statement (24) and have been registered on the International Prospective Register of Systematic Reviews (PROSPERO) with the registered ID: CRD42022367885 on 20 October 2022.

### Search strategy

Two examiners (QP and SY) completed an extensive literature search through online databases independently from 1 December 2019 to 30 October 2022, including MEDLINE, Embase, Web of Science, and Cochrane Central Register of Controlled Trials, with no language restrictions. The search strategy of PubMed was realized by the combination of Medical Subject Headings (MeSH) or free words, including (prone position) and (ARDS or hypoxemic respiratory failure) and (COVID-19 or SARS-CoV-2). The detail of the retrieval strategy is listed in Appendix 1 of the Supplementary material. The search strategies of different databases were adjusted according to the specific situation.

### Study selection

After the completion of the literature search, all duplicate studies were deleted, and then two examiners independently reviewed the studies according to the inclusion and exclusion criteria, which were established according to the PICOS principle (25).

Inclusion criteria: (1) population (P): The study population was COVID-19 patients with ARDS or AHRF, age≥18 years old, (2) intervention (I): awake prone position, (3) comparator (C): to compare the difference in clinical outcomes between patients in the prone position and usual care groups, (4) outcome (O): Primary outcomes included oxygenation, intubation rate, and secondary outcomes included mortality, hospital length of stay, ICU admission, and incidence of adverse events, and (5) study design (S): RCTs.

Exclusion criteria: (1) review, meta-analysis, experimental protocol, case report, and observational study, (2) study on the intervention of intubated patients in the prone position, (3) did not report the outcomes we need, (4) insufficient data or not available through calculation, and (5) non-randomized controlled trials.

### Data extraction and quality assessment

Two examiners extracted data according to the form designed for this systematic review independently and then checked it by the third inspector to ensure accuracy and completeness. The data extracted from the inclusion study included first author, year, study design, study setting, participant characteristics, oxygen delivery, outcomes, and conclusions (Table 1).

**Table 1:** Baseline characteristics of included studies.

**References**	**Study design**	**Study setting**	**Participant characteristics**	**Oxygen delivery**	**Outcome measures**	**Conclusion**
1. Gad (32)	RCT	Single center	*N* = 15: Awake prone position group	NIV	P/F, intubation, mortality, hospital LOS	Prone positioning and NIV showed marked improvement in PaO_2_ and SpO_2_ in COVID-19 patients. In comparing both groups were decreased the rate of conversation of sever COVID 19 to critically ill and avoid invasive ventilation with no significant difference between the two groups
			Age (years): 49 (38–26)	Face mask		
			*N* = 15: Non-invasive ventilation group			
			Age (years): 46 (33–51)			
2. Kharat et al. (33)	RCT	Single center	*N* = 10: Self-prone positioning group	NC	S/F	Self-prone positioning in patients with COVID-19 pneumonia requiring low-flow oxygen therapy showed a reduction of oxygen needs, which did not reach statistical significance, probably due to a small sample size and insufficient statistical power
			Age (years): 54 ± 14			
			*N* = 17: Usual care group			
			Age (years): 60 ± 11			
3. Johnson et al. (34)	RCT	Single center	*N* = 15: Prone positioning group	NC	P/F, intubation, mortality, hospital LOS	patient-directed prone ositioning is not feasible in spontaneously breathing, onintubated atients hospitalized with COVID-19. No improvements in xygenation were observed at 72 or 48 h
			Age (years): 52 (40–65)	HFNC		
			*N* = 15: Usual care group	RA		
			Age (years): 62 (49–75)			
4. Taylor et al. (35)	RCT	Single center	*N* = 27: Awake pone psitioning srategy group	NC HFNC	S/F, intubation, hospital LOS, averse events	Patients in the usual care group had amedian nadir S/F ratio over the 48-h study period of 216 (95% CI, 95–303) vs. 253 (95% CI, 197–267) in the awake pone psitioning srategy group (intraclass correlation coefficient, r = 0.11;95% CI, 0.05–0.18)
			Age (years): 56 (45–66)	RA		
			*N* = 13: Usual care group			
			Age (years): 60 (54–63)			
5. Rosén et al. (36)	RCT	Multicenter	*N* = 36: Prone group	HFNC	Intubation, mortality, hospital LOS, averse events	The implemented protocol for prone position and standard care among patients with hypoxemic respiratory failure due to COVID-19 was safe and increased the duration of prone position, but did not reduce the rate of endotracheal intubation compared with standard care alone
			Age (years): 66 (53–74)	NIV		
			*N* = 39: Control group			
			Age (years): 65 (55–70)			
6. Jayakumar et al. (37)	RCT	Multicenter	*N* = 30: Prone group	HFNC	P/F, intubation, mortality, averse events	There was no significant difference in the cumulative fluid balance, length of stay, respiratory escalation, other medications use or mortality between the groups
			Age (years): 54.8 ± 11.1	NIV		
			*N* = 30: Standard care group	Face mask		
			Age (years): 57.3 ± 12.1			
7. Ehrmann et al. (38)	RCT	Multicenter	*N* = 564: Awake prone positioning group	HFNC	Intubation, mortality, hospital LOS, averse events	Awake prone positioning of patients with hypoxaemic respiratory failure due to COVID-19 reduces the incidence of treatment failure and had a favorable effect on the primary composite outcome of intubation or death within 28 days of enrolment
			Age (years): 61.5 ± 13.3			
			*N* = 557: Standard care group			
			Age (years): 60.7 ± 14.0			
8. Hashemian et al. (40)	RCT	Single center	*N* = 45: Awake prone positioning group	NIV	P/F, intubation, mortality	The application of NIV combined with PP resulted in a significantly shorter length of ICU admission. The need for intubation and the rate of mortality were though lower in the NIV+PP group, and failed to reach the statistical significance
			Age (years): Described in scope			
			*N* = 30: Control group			
			Age (years): Described in scope			
9. Agarwal et al. (20)	RCT	Multicenter	*N* = 205: Awake prone positioning group *N* = 195: Usual care group Total mean age: 58 years	Needed ≥40% oxygen or non-invasive positive pressure ventilation	Intubation, mortality, averse events	In COVID-19 acute hypoxemia, awake prone positioning vs. usual care did not reduce intubation at 30 days
10. Alhazzani et al. (21)	RCT	Multicenter	*N* = 205: Awake prone positioning group	Low- or high-flow oxygen, non-invasive positive pressure ventilation	S/F, intubation, mortality, averse events	In patients with acute hypoxemic respiratory failure from COVID-19, prone positioning, compared with usual care without prone positioning, did not significantly reduce endotracheal intubation at 30 days
			Age (years): 56.8 ± 12.5			
			*N* = 195: Usual care group			
			Age (years): 58.3 ± 13.2			
11. Rampon et al. (22)	RCT	Multicenter	*N* = 159: Self-prone positioning group	NC	Intubation, mortality, hospital LOS, ICU transfer	The study was underpowered to make conclusions regarding the effectiveness of self-prone positioning recommendations and instructions or self-prone positioning itself in reducing clinical deterioration
			Age (years): 52 (39–62)	HFNC		
			*N* = 134: Usual care group	Face mask		
			Age (years): 54 (43–63)			
12. Fralick et al. (39)	RCT	Multicenter	*N* = 126: Prone positioning group	NC	S/F, mortality, hospital LOS, averse events	The change in the ratio of oxygen saturation to fraction of inspired oxygen after 72 h was similar for patients randomized to prone positioning and standard of care
			Age (years): 59.5 (45–68)	HFNC		
			*N* = 122: Standard care group	Face mask		
			Age (years): 54 (44–62)			
13. Ibarra-Estrada et al. (23)	RCT	Multicenter	*N* = 10: Awake prone positioning group	HFNC	S/F, intubation, mortality, hospital LOS, averse events	Awake prone positioning reduced intubation rate and hospital length of stay among patients with COVID-19-induced acute hypoxemic respiratory failure requiring HFNC support, as compared with standard care
			Mean age (years): 58.6 ± 15.8			
			*N* = 10: Standard care group			
			Age (years): 58.2 ± 15.8			
14. Harris and Hamad Medical Corporation (41) (NCT04853979)	RCT	Multicentre	*N* = 10: Awake prone positioning group	HFNC NIV	S/F, intubation, mortality, hospital LOS, averse events	NR
			Mean age (years): NR			
			*N* = 30: Control group			
			Mean age (years): NR			

RCT, randomized controlled trial; NIV, non-invasive ventilation; NC, nasal cannula; HFNC, high-flow nasal cannula; RA, room air; P/F, PaO_2_/FiO_2_ ratio; S/F, SpO2/FiO2 ratio; LOS, length of stay; NR, not reported.

The Cochrane Collaboration Risk of Bias tool (26) was used to assess the quality of included RCTs. This tool assesses bias risk through seven aspects, including random sequence generation, allocation concealment, blinding of participants or personnel, blinding of outcome assessment, incomplete outcome data, selective reporting, and other biases. Each potential source of bias was classified as high, low, or unclear. All divergences in the process of data extraction and quality assessment were resolved through discussions among the three reviewers.

### Data analysis

The oxygenation outcomes and hospital LOS were continuous. Intubation rate, mortality, incidence of ICU admission, and adverse events were dichotomous. We used mean difference (MD) to evaluate continuous outcomes and odds ratio (OR) to evaluate dichotomous outcomes. In continuous outcomes, the median/inter-quartile range (IQR) is converted to the mean and standard deviation by statistical formula if the mean and standard deviation are not available (27, 28). GetData Graph Digitizer 2.26 was used to extract mean values and standard deviations when data exist in the form of figures or charts. We use the method reported by the Cochrane Handbook to calculate the mean and standard deviation of baseline changes (29).

This systematic review compared the effects of prone position and usual care on acute hypoxic respiratory failure in patients with COVID-19. All the included studies were homogeneous. Meta-analysis was carried out using Review Manager 5.4 software (version 5.4 Cochrane Collaboration), and the results were presented in the form of forest plots. The continuous outcomes used inverse variance (IV), and the dichotomous outcomes used Mantel–Haenszel (M–H) to calculate the overall effect with a 95% confidence interval (CI). I^2^ was used to assess heterogeneity between studies, I^2^ < 50% is considered low heterogeneity and I^2^ > 50% was considered moderate to high heterogeneity (30). The fixed effect model was used for low heterogeneity, and the random effect model was used for moderate to high heterogeneity (31). The threshold for significance for *p*-values was 0.05.

## Results

### Study selection and study characteristics

**Figure 1:**
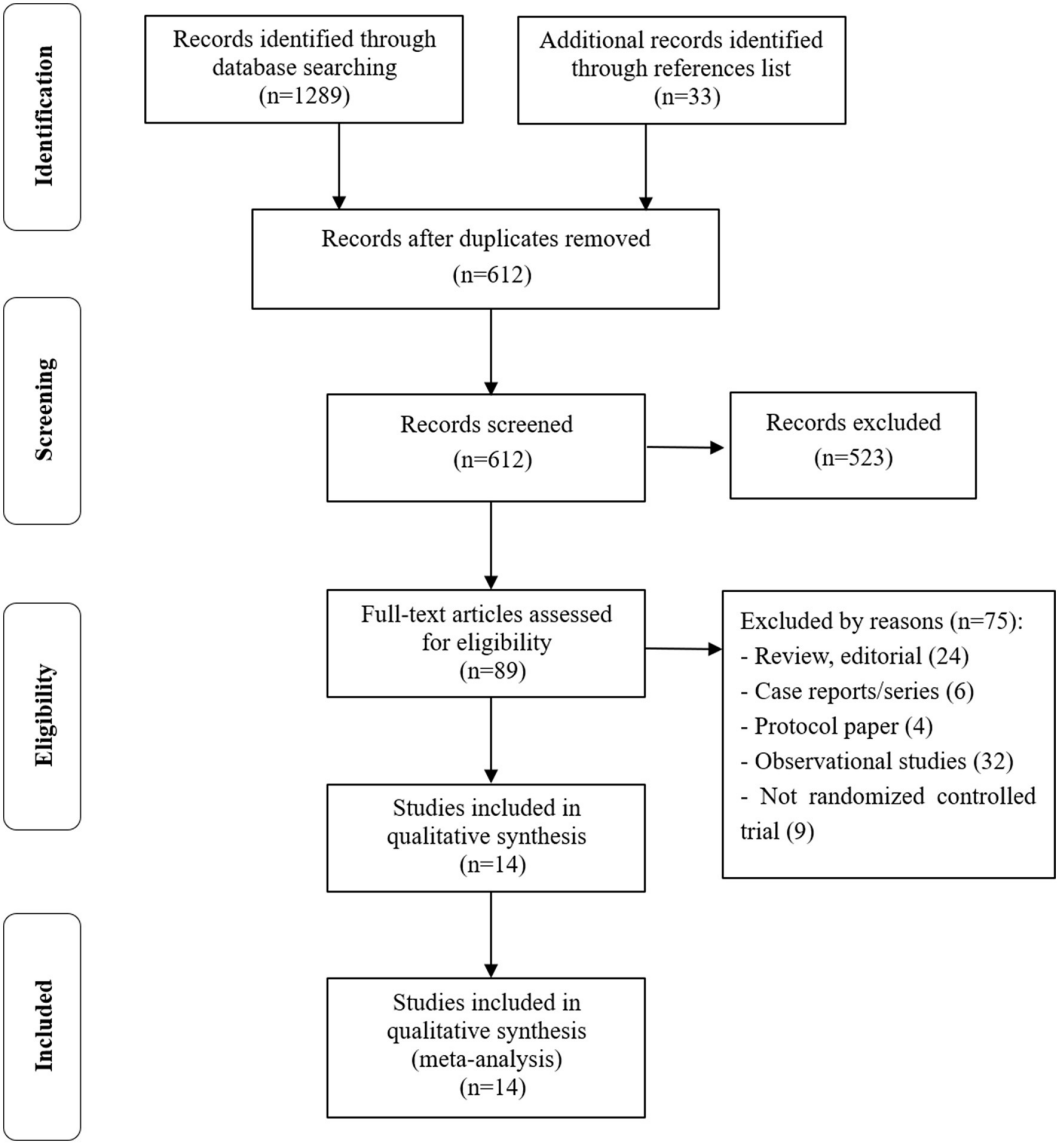
Flow diagram for the study selection process.

After the study search was completed and all duplicates were deleted, a total of 612 studies entered the screening process, and 14 studies and a total of 3,290 patients were finally included in this meta-analysis (20–23, 32–40) (NCT04853979). The process of study screening is shown in Figure 1. All 14 included studies were RCTs, five single-center studies, and nine multi-center studies. They have explored the effect of the prone position on ARDS or AHRF in patients with COVID-19. No incomplete or selective results were reported in the included RCTs, and all the characteristics and data information are presented in Table 1.

### Risk of bias assessment

**Figure 2:**
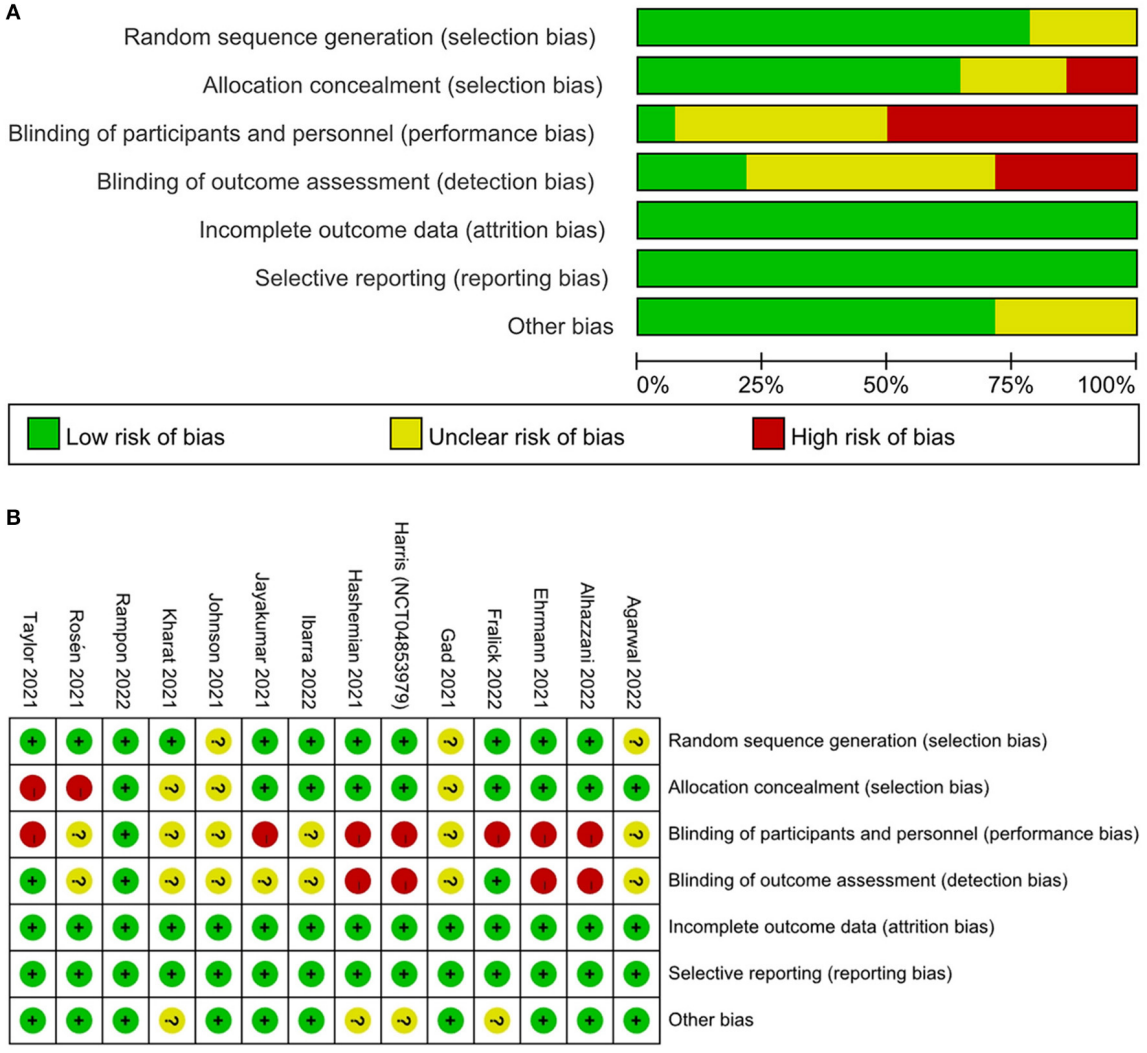
Assessment of risk of bias based on the Cochrane risk of bias tool. **(A)** Risk of bias graph; **(B)** Risk of bias summary.

Eight of the 14 studies were considered to have a high risk of bias (21, 35–39). Two studies showed a high risk of bias in the allocation concealment (35, 36), and seven studies showed a high risk of bias in the blinding of participants or researchers (21, 35, 37–39). The rest of the studies were assessed as low bias risk because they had low bias risk in almost all areas. The result of the assessment of bias risk is shown in Figures 2A, B.

### Oxygenation outcome

**Figure 3:**
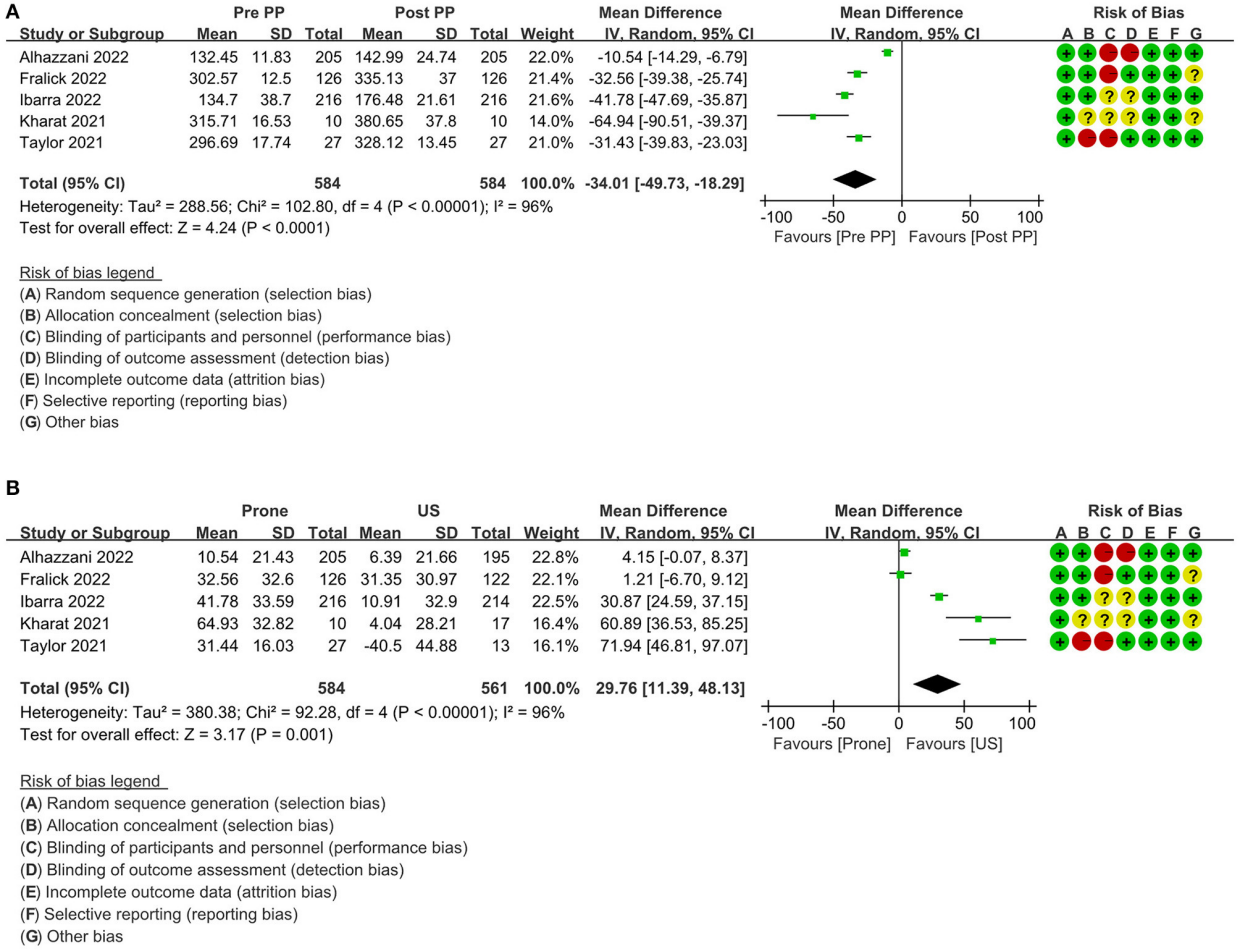
Primary outcome: forest plot of SpO_2_/FiO_2_ ratio in the random-effects model. **(A)** pre-PP vs. post PP; **(B)** prone vs. US; PP, prone position; US, usual care; SD, standard deviation; IV, inverse variance; CI, confidence interval.

Five studies including 1,145 patients reported the SpO_2_/FiO_2_ (S/F) ratio before and after the prone position (21, 23, 33, 35, 39), and the results of the meta-analysis showed that there is a significant difference between them (Figure 3A), MD = −34.01 (95% CI: −49.73 to −18.29; *P* < 0.0001), indicating that prone position can significantly improve S/F ratio in patients with COVID-19 with ARDS or AHRF. I^2^ = 96% indicated that there is a high heterogeneity among studies.

The baseline change data of the S/F ratio between the prone position and usual care group can be obtained from the same five studies (21, 23, 33, 35, 39). The summary results showed that there is a significant difference between the two groups (Figure 3B), MD = 29.76 (95% CI: 11.39–48.13; *P* = 0.001). The results found that the prone position can significantly improve the S/F ratio of patients with COVID-19 compared with the usual care group. I^2^ = 96% also showed high heterogeneity among studies.

### Intubation

**Figure 4:**
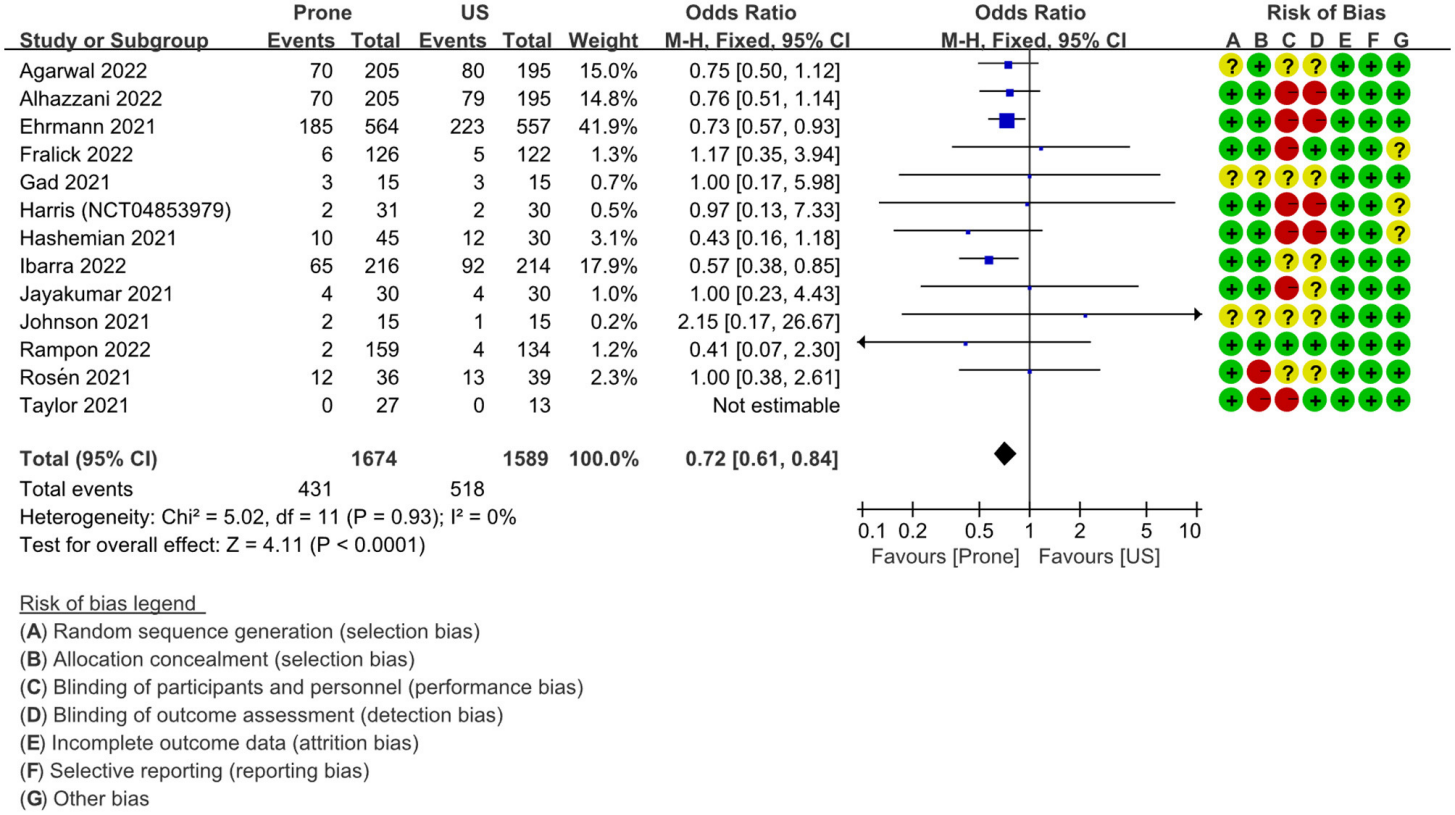
Primary outcome: forest plot of intubation rate in fixed effects model. US, usual care; M–H, Mantel–Haenszel; CI, confidence interval.

A total of 13 RCTs (20–23, 32, 34–40) (NCT04853979) including 3,263 patients, reported the need for intubation between the prone position group and the usual care group, and one study (35) reported that nobody needs to be intubated in both groups. The summary results showed that there is a significant difference between the two groups. Compared with the usual care group, patients had a significantly lower intubation rate in the prone position group, OR = 0.72 (95% CI: 0.61–0.84; *P* < 0.0001; *I*^2^ = 0%) (Figure 4). *I*^2^ = 0% indicated low heterogeneity. In addition, a subgroup analysis of the intubation rate according to the average time of prone position per day (< 8 h >8 h; *P* = 0.18), and ICU vs. non-ICU (*P* = 0.61), there was no significant difference between the two groups (Figures S1, S2 in Supplementary material). On the other hand, the subgroup analysis of the intubation rate according to oxygen delivery shows that the awake prone position group had a significantly lower intubation rate compared with the usual care group in patients with a high-flow nasal cannula (HFNC) or non-invasive ventilation (NIV), OR = 0.65 (95% CI: 0.54–0.78; *P* < 0.00001; I^2^ = 0%), but this difference was not found in the patients with low flow or conventional oxygen therapy (COT), OR = 1.05 (95% CI: 0.59–1.86; *P* = 0.87; I^2^ = 0%) (Figure S3 in Supplementary material).

### Mortality

**Figure 5:**
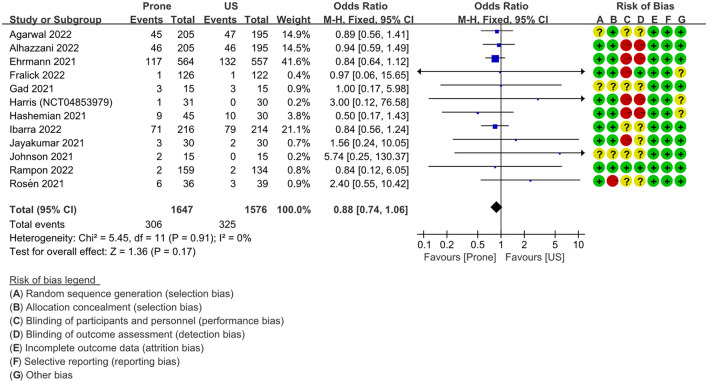
Secondary outcome: forest plot of mortality in fixed effects model. US, usual care; M–H, Mantel–Haenszel; CI, confidence interval.

A comprehensive analysis of the mortality of 3,223 patients in the two groups reported by 10 RCTs (20–23, 32, 34, 36–40) (NCT04853979) showed that the 95% confidence interval of the odds ratio exceeded the limit of no effect, OR = 0.88 (95% CI: 0.74–1.06; *P* = 0.17; I^2^ = 0%), There was no statistically significant difference between the two groups (Figure 5). I^2^ = 0% indicated low heterogeneity.

### Hospital LOS

Eight RCTs (22, 23, 32, 34–36, 38, 39) were reported at the hospital LOS in the prone position and usual care groups, including a total of 2267 patients. The forest plot showed that the 95% confidence interval crosses the threshold of ineffectiveness, and there is no statistical difference between the two groups in hospital LOS, MD = −0.36 (95% CI: −1.39 to 0.66; *P* = 0.49) (Figure S4 in Supplementary material). I^2^ = 98% indicated high heterogeneity.

### ICU admission

Only four RCTs (22, 34–36) reported the incidence of ICU admission including 438 patients, and one study (36) reported that the incidence of ICU admission in the prone position and usual care groups was 75% and 69.23%, respectively. The summary results showed that there is no significant difference between the two groups, OR = 1.20 (95% CI: 0.66–2.19; *P* = 0.55; I^2^ = 0%) as shown in Figure S5 in Supplementary material. I^2^ = 0% indicated low heterogeneity.

### Adverse events

Nine RCTs (20–23, 35–39) including 3,067 patients reported adverse events in the prone position and usual care groups, no adverse events were reported in both groups by Jayakumar et al. (37), and no serious adverse events were reported in all studies. The results showed that there is no significant difference in the incidence of adverse events between the two groups (Figure S6 in Supplementary material), OR = 1.21 (95% CI: 0.58–2.54; *P* = 0.61; I^2^ = 78%). I^2^ = 78% indicated high heterogeneity.

### Publication bias

We used the funnel plot to evaluate the publication bias of several outcomes, including intubation rate, mortality, and ICU admission. The results showed that there is no significant publication bias in the intubation rate and incidence ICU admission rate (Figures S7, S8 in Supplementary material), but there may be publication bias in mortality (Figure S9 in Supplementary material).

## Discussion

Out of all the people hospitalized with COVID-19, 15–30% will go on to develop COVID-19-associated acute respiratory distress syndrome (42). In the supine position, pleural pressure develops along a vertical gradient from the non-dependent to the dependent chest, which is magnified in patients with ARDS (43). Therefore, it is beneficial to carry out the prone position for patients with ARDS.

Our systematic review and meta-analysis confirmed that awake prone position improved oxygenation in COVID-19 patients with ARDS or AHRF significantly compared with usual care, which is consistent with the results of Fazzini et al. (18). The results also found that prone position can reduce the need for intubation, which is consistent with the results of three recent studies (16, 17, 44), but contradicts the results of Fazzini et al. (18), and the reason for this contradiction may be that they have included a large number of observational studies. Although the awake prone position has these advantages, there is likely substantial variation in actual patient adherence and tolerability of the technique. In addition, considering the high risk of clinical deterioration of patients with COVID-19, the awake prone position should be conducted when the patients are in a monitoring state to avoid delaying the timing of intubation (45).

A recent meta-analysis by Weatherald et al. (46) shows that the awake prone position can reduce the need for the intubation of patients with ARDS or AHRF caused by COVID-19, this is consistent with our results, and our subgroup analysis of the intubation rate according to the average time of prone position per day (< 8 h or >8 h), and ICU vs. non-ICU, there was no significant difference between the two groups. Moreover, subgroup analysis of the intubation rate according to oxygen delivery found that the advantage of significantly reducing intubation in the awake prone position was mainly shown in patients receiving HFNC or NIV. The reason may be because patients receiving HFNC or NIV had more severe diseases and greater possibility to progress to endotracheal intubation than patients receiving COT. Weatherald et al. thought that the awake prone position did not significantly improve the oxygenation outcomes; however, by comparing the oxygenation outcomes of patients with COVID-19 before and after the prone position, as well as the oxygenation results of the awake prone position group and usual care group, we found that awake prone position significantly improved the SpO_2_/FiO_2_ ratio of patients with COVID-19. The difference between the two studies may be caused by the heterogeneity of the included studies.

We tried to evaluate the oxygenation outcomes with subgroup analysis according to the average time of prone position, the oxygen delivery methods, and ICU vs. non-ICU, but the existing data do not support us to do so because the number of studies in each group is not enough. All RCTs involved in this meta-analysis showed that the prone position could improve oxygenation in patients with COVID-19 with ARDS or AHRF. The possible mechanisms may be as follows: (i) The prone position reduced the compression of the heart and mediastinum and recruitment of the lungs below the heart, thus improving ventilation (47, 48). (ii) Prone position can reduce the gradient of pleural pressure from the independent area to the dependent region, and make the lung aeration and strain distribution more homogeneous (49–51). The results of a single-center RCT (52) showed that it is beneficial to prolong the prone position of patients with COVID-19. The results of Kaur et al. (53) indicated that the early conscious prone position can reduce mortality in patients with COVID-19 with ARDS or AHRF, which was similar to our results. The research by Vetrugno et al. (54) found that invasive mechanical ventilation increased the risk of barotrauma compared with high-flow nasal oxygen. Therefore, it is meaningful to reduce the intubation rate of patients, and the prone position may help to reduce the barotrauma of patients with ARDS.

Except for two studies (34, 39), other RCTs included in this meta-analysis showed that the prone position could reduce the need for intubation in patients with COVID-19. This finding is of great significance. First, as the number of patients with COVID-19 increases, reducing the need for intubation can alleviate the shortage of medical resources and pressure on the ICU, as well as reduce the risk of aerosol-borne diseases during endotracheal intubation. Second, prolonged intubation may be associated with an increase in mortality (55). Therefore, it is necessary to reduce intubation under the premise of closely observing the progress of the disease. The effect of the prone position may be time-dependent and phase-dependent (56). The subgroup analysis of Kang et al. (17) showed that the intubation rate decreased more significantly in the group with longer prone time. Li et al. (16) found that the prone position has no effect on ICU patients, as the prone position is difficult to reduce the intubation rate of serious patients. However, in our RCTs-based study, there was no difference in intubation rates between the two groups in two subgroup analyses (< 8 h or >8 h; ICU vs. non-ICU).

No significant difference in mortality, hospital LOS, incidence of ICU admission, and adverse events between the prone position and usual care was found in this meta-analysis. This may be caused by almost all studies evaluating them as secondary results or insufficient follow-up time for the patient. In addition, the compliance of patients and the guidance of medical staff may also have an impact on the results. The results of mortality may be affected by publication bias.

## Strengths and limitations

We believed that our study had several following advantages. First, our study evaluates all comparable clinical outcomes comprehensively. Second, we only included RCT studies because the level of evidence of the original study was higher and the results were more convincing.

However, there were also some limitations in our study. Due to the particularity of the awake prone position intervention, some studies were unable to the allocation concealment and blinding of participants or outcome assessment, which may increase the potential risk of bias. In all studies, the start time, duration, and oxygen delivery mode of the prone position were not consistent, which may affect the results. In addition, all studies only reported the results of short-term follow-up, and the long-term prognosis of the patients was unknown. More high-quality RCT studies are needed to analyze the oxygenation outcomes and determine the best start time, duration, and population of prone position in future research. In addition, it is very important to strengthen the guidance to patients and improve their compliance in the prone position for a long time. Future studies should extend the follow-up time and report the long-term prognosis. The effects of related factors such as the degree of dyspnea and the severity of the disease on the results of the study need to be further studied.

## Conclusion

The awake prone position is a promising method for COVID-19 patients with acute hypoxic respiratory failure, with potential benefits including improved oxygenation and intubation rate. There was no significant difference in mortality, hospital length of stay, incidence of ICU admission, and adverse events between the prone position and usual care groups.

## Data availability statement

The original contributions presented in the study are included in the article/Supplementary material, further inquiries can be directed to the corresponding authors.

## Author contributions

QP and SY: protocol/project development, methodology, validation, software, data curation, formal analysis, resources, and writing—original draft. YZ, WZ, MH, BM, HN, and LM: visualization, software, data curation, and formal analysis. LZ, YW, and JY: conceptualization, protocol/project development, visualization, supervision, project administration, funding acquisition, writing—review and editing, and substantial contributions to the conception or design of the work. All the authors are agreed and approved the final manuscript for publication.
