# Erratum: Effects of awake prone position vs. usual care on acute hypoxemic respiratory failure in patients with COVID-19: a systematic review and meta-analysis of randomized controlled trials

**DOI:** 10.3389/fmed.2023.1217614

**Published:** 2023-05-18

**Authors:** Frontiers Production Office

**Affiliations:** Frontiers Media SA, Lausanne, Switzerland

**Keywords:** COVID-19, hypoxemic respiratory failure, ARDS, oxygenation, intubation rate, mortality, awake prone positioning

Due to a production error, authors Yongxiang Wang and Liang Zhang were assigned the incorrect affiliations. Yongxiang Wang should have affiliation 6, and Liang Zhang should have affiliation 1.

The publisher apologizes for this mistake. The original article has been updated.

